# Risk factors for severity of social withdrawal in adolescence: Understanding hikikomori as a spectrum

**DOI:** 10.1192/j.eurpsy.2021.1682

**Published:** 2021-08-13

**Authors:** Y. Hamasaki, T. Nakayama, S. Michikoshi, T. Hikida

**Affiliations:** 1 Faculty Of Contemporary Society, Kyoto Women’s University, kyoto, Japan; 2 Institute For Protein Research, Osaka University, Osaka, Japan

**Keywords:** social withdrawal, Hikikomori, adolescence, risk factor

## Abstract

**Introduction:**

Social withdrawal, or hikikomori, is one of Japan’s most serious psychosocial issues. The concept gained international attention around 2010 and widespread psychiatric epidemiological studies have since been conducted.

**Objectives:**

With an understanding of the extensive range of hikikomori circumstances as a spectrum, we aimed to quantitatively measure the severity of hikikomori in adolescent subjects, an age group considered particularly susceptible to the condition, and to identify factors associated with its severity.

**Methods:**

We selected population demographics, socioeconomic data, and psycho-behavioral characteristics as factors related to hikikomori and explored their associations with hikikomori severity using cross-sectional analysis. Subjects were a patient group of middle school students examined as outpatients at a psychiatric clinic during adolescence for a chief complaint of hikikomori and a control group of middle school students matched for sex and age. Subjects’ parents completed a questionnaire pertaining to their child’s hikikomori symptoms and living environment along with the Child Behavior Checklist (CBCL). The data collected was then statistically analyzed.

**Results:**

T-test results demonstrated that scores for all CBCL syndrome scales were significantly higher in the patient group, but no scores fell within the clinical range. Multiple regression analysis revealed that being anxious/depressed, somatic complaints, lack of communication between parents, and overuse of the Internet were statistical predictors of hikikomori severity.

**Conclusions:**

It may be possible to prevent hikikomori from becoming severe if the above predictors are used to identify high-risk individuals requiring active intervention while hikikomori is at an early stage.

**Disclosure:**

No significant relationships.

